# Intermolecular Interaction of Tetrabutylammonium and Tetrabutylphosphonium Salt Hydrates by Low-Frequency Raman Observation

**DOI:** 10.3390/molecules27154743

**Published:** 2022-07-25

**Authors:** Yasuhiro Miwa, Tomoki Nagahama, Harumi Sato, Atsushi Tani, Kei Takeya

**Affiliations:** 1 Graduate School of Human Development and Environment, Kobe University, 3-11 Tsurukabuto, Nada, Kobe 657-8501, Japan; 186d415d@stu.kobe-u.ac.jp (Y.M.); tomo.n.0820.shu@gmail.com (T.N.); hsato@tiger.kobe-u.ac.jp (H.S.); 2 Institute for Molecular Science (IMS), 38 Nishigonaka, Myodaiji, Okazaki 444-8585, Japan; takeya@ims.ac.jp

**Keywords:** semi-clathrate hydrate, intermolecular interaction, interionic interaction, low-frequency Raman, equilibrium temperature

## Abstract

Semi-clathrate hydrates are attractive heat storage materials because the equilibrium temperatures, located above 0 °C in most cases, can be changed by selecting guest cations and anions. The equilibrium temperatures are influenced by the size and hydrophilicity of guest ions, hydration number, crystal structure, and so on. This indicates that intermolecular and/or interionic interaction in the semi-clathrate hydrates may be related to the variation of the equilibrium temperatures. Therefore, intermolecular and/or interionic interaction in semi-clathrate hydrates with quaternary onium salts was directly observed using low-frequency Raman spectroscopy, a type of terahertz spectroscopy. The results show that Raman peak positions were mostly correlated with the equilibrium temperatures: in the semi-clathrate hydrates with higher equilibrium temperatures, Raman peaks around 65 cm^−1^ appeared at a higher wavenumber and the other Raman peaks at around 200 cm^−1^ appeared at a lower wavenumber. Low-frequency Raman observation is a valuable tool with which to study the equilibrium temperatures in semi-clathrate hydrates.

## 1. Introduction

Semi-clathrate hydrate compounds formed from host water molecules and guest molecules have a similar structure to clathrate hydrate typified by methane hydrate. They have a hydrogen-bonded cage structure that surrounds a guest cation, and one of the water molecules is replaced with a guest anion. Due to having empty cages and mild formation conditions under atmospheric pressure (the equilibrium temperature is above 0 °C), semi-clathrate hydrates have potential for use in gas separation and storage as well as for latent heat storage materials [1,2,3]. In terms of practical use, semi-clathrate hydrates with greater gas storage capacity and phase change at an adaptive temperature are required [4,5], and it is desired to freely select the combination of ions as guest substances [6].

A large number of the guest substances, i.e., combinations of various cations and anions, have been reported [7,8,9,10,11]. Among them, tetrabutylammonium (TBA) salts and tetrabutylphosphonium (TBP) salts, which are quaternary onium salts, are well known as ionic guest substances. In tetrabutylammonium bromide (TBAB) semi-clathrate hydrate, bromide anions displace part of the host water molecules and form cage structures with them. Meanwhile, the butyl groups of TBA cations occupy four partially broken cages. A cage is composed of water molecules in several different patterns, and the total cage structure is defined by the combination of these cages. For this reason, even for the same guest substance, the crystal structure of semi-clathrate hydrate may differ depending on the cage structure of the surrounding water molecules. For example, TBAB hydrates have two main crystal structures depending on hydration number (TBAB·26H_2_O and TBAB·38H_2_O) with different equilibrium temperatures (12.0 and 9.5 °C) and dissociation enthalpies (150.7 and 219.4 kJ/mol), respectively [12,13]. In addition, the hydrates encapsulating guest substances with different combinations of TBA/TBP cations and halide anions (bromide/chloride) have inherent enthalpies and equilibrium temperatures; tetrabutylammonium chloride (TBAC) hydrate has a dissociation enthalpy of 156 kJ/mol and an equilibrium temperature of 15.1 °C [14], and tetrabutylphosphonium chloride (TBPC) hydrate has a dissociation enthalpy of 164.7 kJ/mol and an equilibrium temperature of 10.3 °C [15]. Tetrabutylphosphonium bromide (TBPB) hydrates also have two crystal structures depending on the hydration number (TBPB·32H_2_O and TBPB·38H_2_O) [16,17]. Their dissociation enthalpies are 184.0 kJ/mol for TBPB·32H_2_O and 211.8 kJ/mol for TBPB·38H_2_O, and their equilibrium temperatures are 9.1 and 9.2 °C, respectively [18].

Thus, the equilibrium temperature and the dissociation enthalpy are greatly different in each guest substance. The cause of the difference in the equilibrium temperature between anion species such as bromide and chloride ions is currently estimated to be the size of the ionic radius of the anion [19], that is, the size of the partial molar volume of the anion [20]. Though other inferences about the shape of the anion, the fit of the cation into the cage, and the hydrophilicity have also been considered as reasons for the differences in the equilibrium temperatures [6,8,10,21], no view to understand the difference in equilibrium temperatures from the intermolecular interactions between ions or between guest and host molecules has yet been formulated.

Intermolecular interactions are strongly associated with phase change materials because the change in the binding force directly affects the equilibrium temperatures of materials. The energy of this intermolecular interaction corresponds to the terahertz region (THz) between infrared waves and microwaves. In other words, spectroscopy in the THz region can reveal intermolecular interactions such as lateral and translational vibration binding energies instead of intramolecular bending and stretching vibrations which reflect the conformation of guest molecules. For example, several studies were conducted on the higher order structure of proteins, hydration of polymers, and discrimination of crystal polymorph of pharmaceutical products [22,23,24]. In general, the terahertz region often refers to the 0.1–10 THz range. Low-frequency Raman spectroscopy, known as a type of terahertz spectroscopy, used in this study, covered the region of 3.3–330 cm^−1^ (corresponding to 0.1–10 THz) below the fingerprint region. Practically, intermolecular interactions during phase change were investigated. For example, terahertz spectroscopic observation of carbamazepine revealed that intermolecular interaction changed during solid and glass transitions [25]. In the observation of the change in the low-frequency Raman spectra upon the melting of ion liquids, the structural change of the substances was discussed in terms of intermolecular binding strength [26]. These studies suggest that the direct observation of intermolecular binding forces in semi-clathrate hydrates could provide a correlation with equilibrium temperatures.

The simple structure of clathrate hydrates of Xe hydrate and tetrahydrofuran (THF) hydrate was investigated using low-frequency Raman spectroscopy [27,28,29]. They showed two Raman peaks at around 210 cm^−1^ and below 100 cm^−1^. The peak around 210 cm^−1^ was attributed to the host water framework. The other peak below 100 cm^−1^ was related to the localized anharmonic motions of guest atoms and host lattice phonons, which was discussed in Xe hydrate. In addition, molecular dynamics simulation for methane hydrate estimated the degree of rotation and vibration as translational rattling motions of molecules in the low-frequency region [30]. According to these studies, intermolecular interactions, especially between guest/guest or guest/host molecules in semi-clathrate hydrates, mainly appeared near or below 100 cm^−1^. Nevertheless, semi-clathrate hydrates below 100 cm^−1^ were not investigated, although the Raman spectra of genuine clathrate hydrates were observed below 100 cm^−1^ as described above. In addition, Raman measurements of semi-clathrate hydrates in the THz region of 100–300 cm^−1^ were performed only for TBAB hydrate [31]. Therefore, we investigated the low-frequency Raman spectra, particularly of less than 300 cm^−1^ with several quaternary onium salts and their semi-clathrate hydrates to reveal the intermolecular interaction between guest/guest and guest/host molecules that may have a considerable influence on the equilibrium temperatures.

## 2. Results and Discussion

### 2.1. Semi-Clathrate Hydrates, Clathrate Hydrates, and Ice

**Figure 1:**
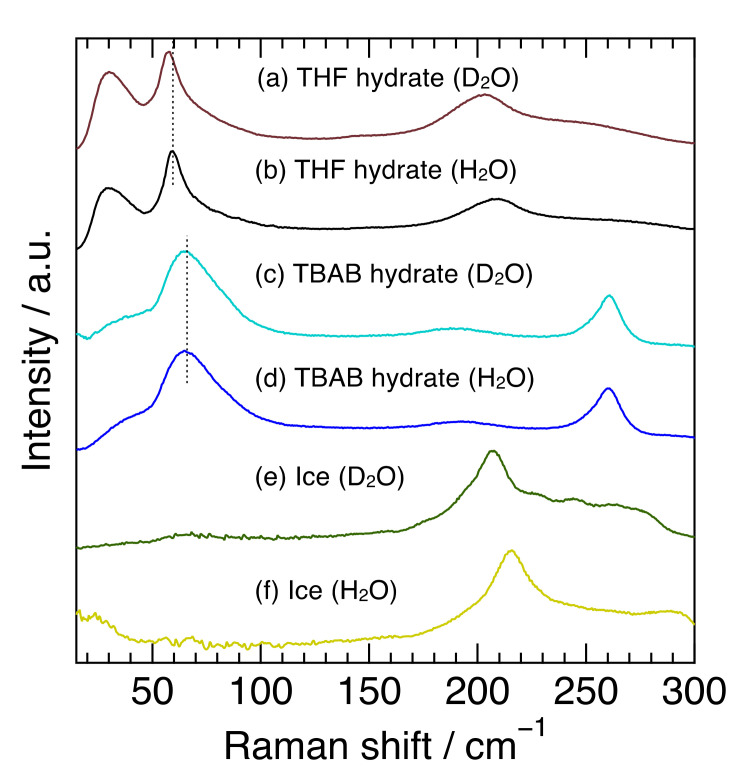
Raman spectra for THF hydrate, TBAB hydrate, and ice with H_2_O or D_2_O. The dotted vertical lines around 65 cm^−1^ from (a–d) were used to make it easier to see a peak shift.

Figure 1 shows the Raman spectra at 263 K in the 15–300 cm^−1^ region of TBAB hydrate (hydration number was 26), THF hydrate and ice with H_2_O or D_2_O. This spectral region contains common or unique features among semi-clathrate hydrate, clathrate hydrate, and ice, all of which are composed of water molecule networks. In the region from 100–300 cm^−1^, the TBAB hydrate (H_2_O) had an apparent peak at 261 cm^−1^ and a broad one around 191 cm^−1^. The peak at 261 cm^−1^ is related to TBA cations and the peak around 191 cm^−1^ is attributed to the intermolecular hydrogen stretching O-O vibration mode, which is in good agreement with the previous Raman investigations [31,32]. TBAB hydrate (D_2_O) revealed a peak at 261 cm^−1^ which was the same position of the TBAB hydrate (H_2_O) and a peak around 189 cm^−1^ which was slightly shifted to a lower wavenumber from 191 cm^−1^ in TBAB hydrate (H_2_O). On the other hand, THF hydrate and ice showed a peak around 200 cm^−1^, reflecting the O-O vibration mode [28]. The peaks in THF hydrate were observed at 208 cm^−1^ for the H_2_O hydrate and 203 cm^−1^ for the D_2_O hydrate and the peaks in ice were observed at 214 cm^−1^ for H_2_O ice and 208 cm^−1^ for D_2_O ice. The peaks in all the deuterated samples around 200 cm^−1^ shifted to a lower wavenumber. The peak around 191 cm^−1^ in TBAB hydrate was much broader than that in THF hydrate and ice. These results indicate that the peak position and broadness of O-O vibration modes apparently depend on the materials with hydrogen-bonded networks and their hydrogen isotope in water molecules.

In the region below 100 cm^−1^, TBAB hydrate (H_2_O) had a broad peak around 67 cm^−1^ and THF hydrate (H_2_O) had a sharp peak at 59 cm^−1^, although no clear peak was observed in ice. The sharp peak of THF hydrate was attributed to the host water framework in structure-II clathrate hydrate [28]. The Raman and inelastic neutron scattering (INS) spectra of Xe hydrate (Structure-I) also have a similar peak at 60 cm^−1^, which is considered to be based on the water framework [27]. Furthermore, the Raman spectrum of liquid water shows weak peaks at 53 and 73 cm^−1^ [33]. Isotope effects on the peak position were also observed in THF hydrate (59.0 cm^−1^ for the H_2_O hydrate and 57.4 cm^−1^ for the D_2_O hydrate), while they were not clearly observed in TBAB hydrate. Though the broader peak in TBAB hydrate might mask the isotope effect, the peak in TBAB hydrate may be attributed to not only the water framework but also to other factors. Subbotin et al. reported that the phonon density of states in this region was different between semi-clathrate hydrate and genuine clathrate hydrate [34]. Furthermore, the shift of the peak position around 67 cm^−1^ was not observed between 173 K and 273 K, although the peak near 191 cm^−1^, intermolecular hydrogen stretching O-O vibration mode, shifted to a higher wavenumber with decreasing temperature. These results reinforce that factors other than the water molecule framework itself affected the characteristics of the peak around 67 cm^−1^ in TBAB hydrate.

**Figure 2:**
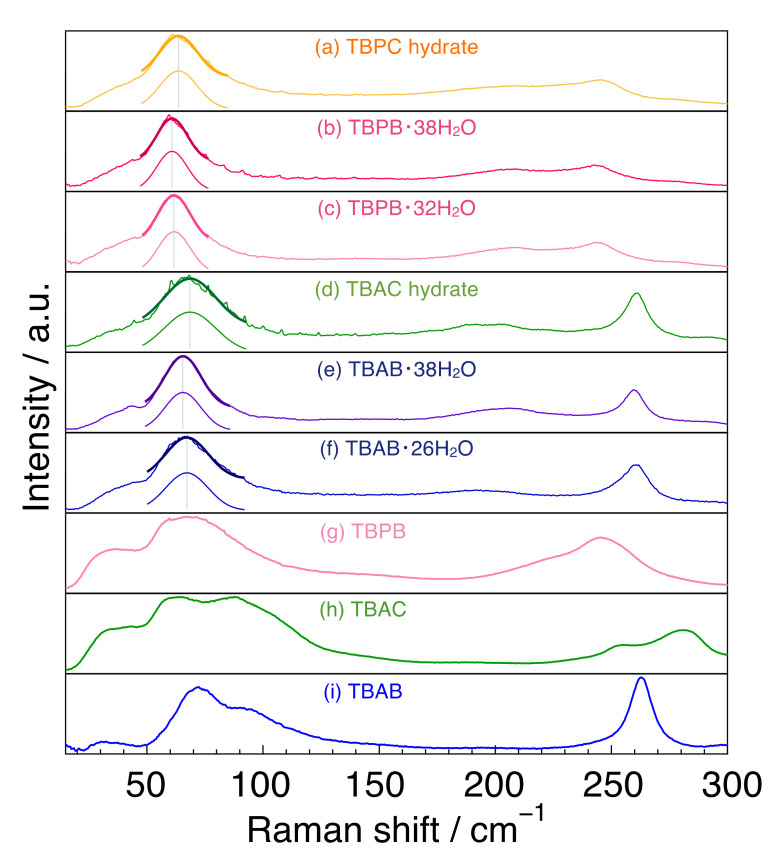
Raman spectra for tetrabutylammonium and tetrabutylphosphonium salts and their hydrates. (a) TBPC hydrate; (b) TBPB·38H_2_O; (c) TBPB·32H_2_O; (d) TBAC hydrate; (e) TBAB·38H_2_O; (f) TBAB·26H_2_O; (g) TBPB; (h) TBAC and (i) TBAB. The solid thick lines around 65 cm^−1^ are single peak spectra simulated by fitting.

The Raman spectra of tetrabutylammonium and tetrabutylphosphonium salts and their hydrates (H_2_O) are summarized in Figure 2. For the spectra of the hydrates, single peak spectra around 65 cm^−1^ were simulated by fitting and are indicated in Figure 2.

### 2.2. Tetrabutylammonium and Tetrabutylphosphonium Salts

First, we focused on the spectra of the three salts. In the region of 150–300 cm^−1^, TBAB had only one peak at 263 cm^−1^, whereas TBPB and TBAC had two peaks at around 220 cm^−1^ and at 247 cm^−1^ in TBPB and at 255 and 280 cm^−1^ in TBAC. In the 50–150 cm^−1^ region, undistinguished peaks were observed in all the salt samples. TBAB had a main peak at 71 cm^−1^ and another peak around 92 cm^−1^ with about half of the intensity of the main peak. These two peaks had a similar line width. TBAC had broad peaks around 59 and 83 cm^−1^ together with a smaller peak at around 110 cm^−1^, and TBPB had broad peak(s) around 68 cm^−1^.

The two main peaks in TBAC were a little far apart from each other in comparison with those in TBAB and TBPB, which was possibly caused by the mobility of anion [35]. Besides, the peak intensity around 83 cm^−1^ in TBAC was comparatively large rather than that around 92 cm^−1^ in TBAB, which was also considered to be due to the difference of anions. Similar trends were also observed in the THz infrared absorption spectra in the liquid state of TBAB and TBAC [36]. Quantum calculations for TBAB in this wavenumber region revealed four main infrared active peaks at 46, 57, 80, and 93 cm^−1^ and corresponding Raman active peaks at similar positions [37]. In comparison with the simulated Raman peaks, the peak positions observed in this study were slightly shifted to lower wavenumber in all cases. It is noteworthy that the clearly visible peak around 70 cm^−1^ was attributed to the translation of the anion along with a degree of flexing and rotation of the alkyl chains, related to the cation–anion interaction [37].

### 2.3. Tetrabutylammonium and Tetrabutylphosphonium Salt Hydrates: Around 240–265 cm^−1^

In the range of 240–265 cm^−1^, TBAB and TBAC hydrates had a sharp peak at 261 cm^−1^, whereas TBPB and TBPC hydrates had a broad peak around 245 cm^−1^. The peak in TBA hydrates was attributed to a stretching lattice vibration mode in the cation [38], indicating that the peak in TBP hydrates is caused by the same motion. The sharp peak of the TBA ion may be due to the fact that the TBA ion has only a trans conformation, while the TBP ion has both trans and gauche conformations, as reported by Kobori et al. who investigated the conformations in the comparison of tetrabutylphosphonium hydroxide (TBPOH) hydrate and tetrabutylammonium fluoride (TBAF) hydrate [20]. In addition, Muromachi et al. [17] reported that TBP ions distorted the cage structure by pushing the cage more strongly than TBA ions because of the larger bond length between carbon and phosphonium than that between carbon and nitrogen. This is another reason for the broader peak observed in TBP hydrate.

### 2.4. Tetrabutylammonium and Tetrabutylphosphonium Salt Hydrates: Around 200 cm^−1^

All peaks were weak and broad in comparison with those in the THF hydrate and ice as shown in Figure 1. Peak tops appeared at 193 cm^−1^ for TBAB·26H_2_O, 195 cm^−1^ for TBAC hydrate, 203 cm^−1^ for TBAB·38H_2_O, 205 cm^−1^ for TBPC hydrate, 208 cm^−1^ for both TBPB·32H_2_O and TBPB·38H_2_O. The peak positions of the TBA salt hydrates were at a lower wavenumber than those of the TBP ones. Besides, the TBAB hydrate with higher hydration number (TBAB·38H_2_O) had a peak at a higher wavenumber than TBAB·26H_2_O, although TBPB hydrates with different hydration numbers had peaks at the same position. As described before, these peaks around 200 cm^−1^ were attributed to intermolecular hydrogen stretching O-O vibration mode. In comparison with the peaks in liquid water and solid ice, all peaks in the semi-clathrate hydrates were observed between the peaks of liquid water (175 cm^−1^) and solid ice (213 cm^−1^) [33,39].

The differences in the peak positions were first considered in terms of the bonding distance of the water framework. The neighbor distance between oxygen atoms in water molecules was obtained for the three following hydrates using the reported crystal structures [14,17,40]. The average O-O length is 2.76 Å for TBAB·38H_2_O and TBPB·38H_2_O, whereas it is 2.80 Å for TBAC·30H_2_O, which is slightly longer than that in the bromide hydrates. The variation of the neighbor O-O length in TBAC hydrate was also larger than that in the two bromide hydrates. According to reference [41], the distribution of the O-O length in solid ice is concentrated between 2.7 and 2.8 Å with a maximum at 2.76 Å, whereas the distribution in liquid water is much broader (2.5–3.2 Å) with a longer average distance (2.81 Å). Though the average O-O lengths in these bromide hydrates were closer to that in the solid ice, the observed peaks in the hydrates appeared around 200 cm^−1^, which is a lower wavenumber than for ice. Similarly, though the average O-O length in TBAC·30H_2_O was closer to that in the liquid water, the observed peak was around 195 cm^−1^, with a higher wavenumber than the liquid water peak. These results mean that this perspective is insufficient to explain the differences of the peak position around 200 cm^−1^ among the hydrates.

Then, we focused on the tetrahedrality of water molecules in the hydrates. The peak position in this region was reflected by the degree of deformation of the tetrahedral unit and the shift to a lower wavenumber indicated a deformation of the tetrahedral unit [42]. All the peaks in the hydrates were located at a lower wavenumber than that in solid ice, indicating that the tetrahedrality of water molecules deformed in the hydrates rather than in ice. In comparison with the TBA and TBP salt hydrates, peaks at lower wavenumbers in the TBA hydrates than in the TBP hydrates suggested that tetrahedrality was more deformed in the TBA hydrates. In particular, TBAB·26H_2_O and TBAC hydrates, whose peaks were located below 200 cm^−1^, could be highly deformed. The deformation of the tetrahedral units is considered to be related to the presence of soft hydrogen bonds, i.e., water-like hydrogen bonds [31]. This could be the reason why TBAB·26H_2_O and TBAC hydrates had relatively higher equilibrium temperature.

The small difference in the peak positions near 200 cm^−1^ in the hydrates could be also affected by the charge distribution of onium ions because the motion of water molecules in the hydrates was influenced by the charge distribution. Based on molecular orbital simulation [21], the charge density on phosphonium was concentrated in the TBP ion rather than that of nitrogen in the TBA ion. In other words, the positive charge in the TBA ion was distributed compared to the TBP ion. This implies that the water molecules surrounding the center cation interact with phosphonium and move less easily in TBP hydrates. This may also cause the peaks near 200 cm^−1^, at a relatively high wavenumber in the TBP hydrates.

All these results indicate that the vibrational peaks between water molecules in the hydrates could be used to discuss the water molecule network including influences by guest cations, although the intensity of the peaks themselves was smaller than that of ice or clathrate hydrate. In contrast, the effect of guest anions was less pronounced.

### 2.5. Tetrabutylammonium and Tetrabutylphosphonium Salt Hydrates: Below 100 cm^−1^

**Figure 3:**
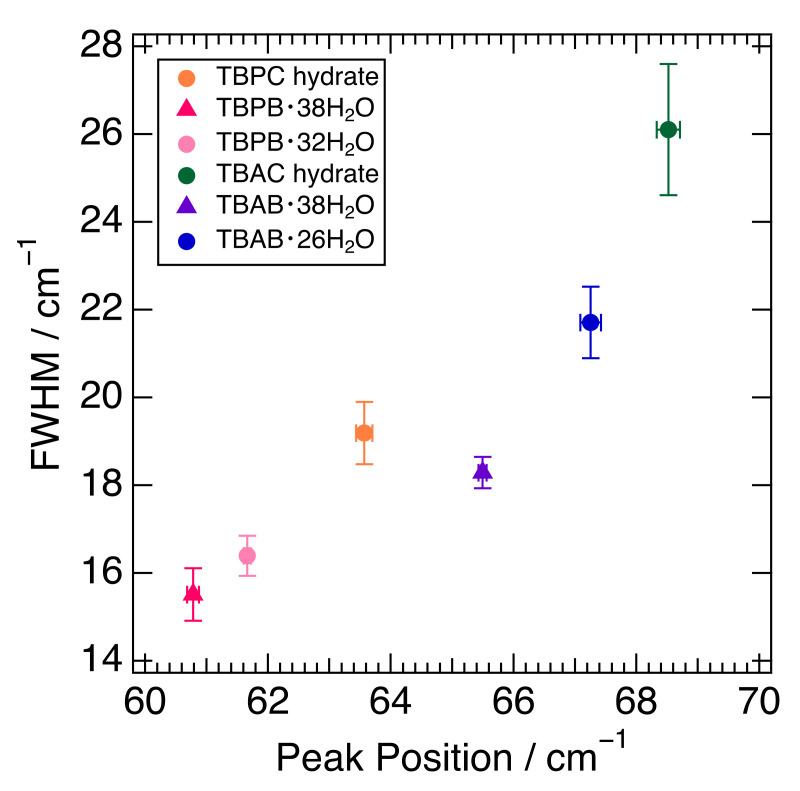
Relationship between peak position and FWHM in six semi-clathrate hydrates. Markers are explained in the legend.

The fitting results of the peak position around 65 cm^−1^ and their full width at half maximum (FWHM) in six semi-clathrate hydrates are summed up in Figure 3. The peaks of the TBA series are located at about 65–69 cm^−1^ and those of the TBP series at 61–64 cm^−1^. The FWHM of the TBP series was relatively narrower than that of the TBA series. TBAC and TBPC hydrates had a wider FWHM than TBAB and TBPB hydrates, respectively. TBAB·26H_2_O had a wider FWHM than TBAB·38H_2_O, which is related to the lower hydration number. Similar results were observed in TBPB·32H_2_O and TBPB·38H_2_O. As discussed in Section 2.1, the peak around 65 cm^−1^ in TBAB·26H_2_O was attributed to not only the water molecule framework but also other factors such as guest anions and cations. This is supported by the variation of the peak position and FWHM in six semi-clathrate hydrates as shown in Figure 3.

Figure 3 shows that the differences in the peak positions largely depend on the cations. In the comparison of TBAB·38H_2_O and TBPB·38H_2_O, which have the same crystal structure and hydration number, the interionic distance of TBAB was 5.703 Å [40] and closer than that of TBPB, 5.837 Å [17]. This is one of the reasons why TBA salt hydrates have peaks at relatively higher wavenumbers. Although another reason may be a lower mass in the ammonium ion than in the phosphonium one, the difference is small and would not affect the peak position effectively. In addition, one of the more intriguing factors is the charge dispersion of the onium ions. As mentioned above, the charge distribution of TBA ions was relatively delocalized compared to that of TBP ions. This indicates that the cation–anion interaction was expected to be stronger in the TBA salt hydrates because a study on quaternary phosphonium/ammonium ionic liquids (ILs) showed that the cation–anion interaction was stronger in the dispersive ammonium ILs than in the phosphonium ILs [43]. This also causes the peaks in TBA salt hydrates at a comparatively higher wavenumber.

The influence of the anions may also have been observed in this region due to the mass of the anions; among the same cations, the hydrates with Cl ions showed peaks at a higher wavenumber than those with Br ions. The smaller ionic radius of the Cl ion is also responsible for the shorter interionic distance, which may affect the peak position at a higher wavenumber. Since the anions were incorporated into the water molecule network, the interionic interaction affected the stability of the cages. This implies that the peaks related to interionic interaction at a higher wavenumber are related to a higher equilibrium temperature. It is consistent with the fact that the hydrates with Cl ions have relatively high equilibrium temperatures among the same cations as shown in Section 1.

A peak shift to a low wavenumber was observed around 65 cm^−1^ in the hydrates in comparison with the salt samples. This peak shift was due to hydration, which is in agreement with several studies using the terahertz region [44,45]. The cations were isolated by the hydrogen bonding of water molecules, which increased the interionic distance and lowered the binding energy. In the case of TBAB, the interionic distance actually increased from 4.905 Å [46] for TBAB to 5.703 Å [40] for TBAB 38H_2_O. Another reason may be that hydration restricts the motion of the ions. Therefore, the spectra observed in the hydrates were generally similar without the significant differences observed between the ILs of TBAB and TBAC. The ratio of the average frequency of the peak positions in TBAB and TBAC ILs is 0.70 [36].

As for the differences in FWHM, crystal structures and hydration numbers affect the FWHM; tetragonal bromide hydrate, TBAB·26H_2_O, expressed a wider peak than the orthorhombic hydrate, TBAB·38H_2_O, and the hydrate with a lower hydration number, TBPB·32H_2_O, had a wider peak than that with a higher hydration number, TBPB·38H_2_O. This is probably due to the fact that the higher hydration number in the orthorhombic hydrates reduced the number of ionic guests per unit cell [8], i.e., the influence of cation–anion interactions. It was noted that the FWHM was even smaller for THF hydrate (clathrate hydrate), which had no ionic interaction as shown in Figure 1. For further investigation, a simulation study was necessary to clarify the main causes behind the differences in FWHM.

### 2.6. Raman Peaks and Equilibrium Temperatures in Semi-Clathrate Hydrates

Low-frequency Raman observation of the six semi-clathrate hydrates revealed the peak shift was related with interionic distance, hydration number, and guest ion species. We found the trend that for a higher hydration number, with bromide ion as an anion, and/or TBP ion as a cation, (a) Raman peaks of around 65 cm^−1^ shifted to a lower wavenumber, (b) Raman peaks around 200 cm^−1^ shifted to a higher wavenumber, and (c) Raman peaks around 65 cm^−1^ became narrower. The positions of these peaks were generally inversely correlated. This may be because the peak around 200 cm^−1^ mainly reflected the interaction between water molecules and the peak around 65 cm^−1^ mainly reflected the interaction between the ions.

Interestingly, in most cases of the semi-clathrate hydrates with a higher equilibrium temperature among all the samples, (a) Raman peaks of around 65 cm^−1^ appeared at a higher wavenumber and a (b) Raman peak around 200 cm^−1^ appeared at a lower wavenumber. This means that low-frequency Raman observations are valuable as a tool to study the equilibrium temperature in semi-clathrate hydrates. For further investigation from the physicochemical aspect, molecular simulation should be performed to understand the attribution of the peaks at the low-frequency region.

## 3. Materials and Methods

### 3.1. Preparation of Semi-Clathrate Hydrates

The chemicals used in this study are listed in Table 1. According to the previous reports, the aqueous solutions were prepared with a composition in the range of mass percent concentration *w* = 0.32–0.40 (*w* = 0.40 for TBAB·26H_2_O [12], *w* = 0.32 for TBAB·38H_2_O [13], *w* = 0.34 for TBAC hydrate [14], *w* = 0.37 for TBPB·32H_2_O [47], *w* = 0.30 for TBPB·38H_2_O [17], *w* = 0.36 for TBPC hydrate [15]) using ultrapure water. Once the initial crystal nuclei were formed by cooling the aqueous solutions at −20 °C, semi-clathrate hydrates were formed by maintaining the samples at 3 °C for at least 3 days. THF hydrate was also prepared as a reference of a genuine clathrate hydrate by the same procedure using an THF aqueous solution adjusted to *w* = 0.19. In addition, to observe the isotope effect, TBAB hydrate (hydration number was 26) and THF hydrates with D_2_O were prepared according to the same procedure using the aqueous solutions with the same molar ratio as those of H_2_O. The salt samples shown in Table 1 were also prepared by keeping them dry with nitrogen gas to avoid further hydration just before Raman measurements.

Two observation methods of Raman and equilibrium temperature were used to confirm the hydrate samples had the desired hydration number, especially for the different hydration numbers of TBAB hydrates (26H_2_O and 38H_2_O) and TBPB ones (32H_2_O and 38H_2_O). For TBAB hydrates, based on the previous studies on the ordinary Raman spectra of the hydrates [31], we confirmed the hydration number of 26 or 38. We observed slightly different spectra both in the C-H stretching modes of the butyl groups (2700–3050 cm^−1^) and in CH_2_ bending modes of the alkyl groups (around 1320 and 1455 cm^−1^), which were comparable to those in the previous study [31]. For TBPB hydrates, we confirmed the hydration numbers using the equilibrium temperature observed by increasing the temperature of the thermostatic bath carefully, because no clear differences in the Raman spectra emerged. The other hydrates were also recognized as the desired semi-clathrate hydrates by the same method.

### 3.2. Low-Frequency Raman Measurements

For the Raman scattering study, the particle samples were placed into aluminum pans on the temperature control stage (THMS600, Linkam, Salfords, UK) with liquid nitrogen. The semi-clathrate hydrates were measured at a temperature in the range of 173–273 K. The THF hydrates and water ices were measured only at 263 K. The salt samples were measured at room temperature.

Low-frequency Raman measurements were performed using LabRAM HR Evolution (HORIBA, Kyoto, Japan). The wavenumber calibration was performed on a Si plate with a Raman line at 520.7 cm^−1^. The spectral resolution was 0.5 cm^−1^ and wavelength of the laser was 532 nm. The laser beam had a power of about 100 mW. Scans were performed 10 times and the exposure time was 15 s. The background noise was separately acquired and subtracted from the original spectrum to obtain the Raman spectrum from the samples.

### 3.3. Spectrum Processing

For discussion, the observed spectrum was divided into two wavenumber ranges of below 100 cm^−1^ and 100–300 cm^−1^. The common features of the peaks below 100 cm^−1^ in the semi-clathrate hydrates were a large broad peak at around 65 cm^−1^ and a tiny broad one below 50 cm^−1^. The weak peak below 50 cm^−1^ appeared on the tilt background and showed no apparent peak top, meaning that it was difficult to identify the peak accurately. Therefore, we focused on the peak around 65 cm^−1^ in further discussions. To evaluate the peak position and FWHM, we assumed the constant base line from 47–50 cm^−1^ to 80–90 cm^−1^ to decrease the influence from the broad peak below 50 cm^−1^ and performed fitting with a single gaussian peak after subtraction with the base line. We attempted to evaluate peak position and FWHM with several different base lines, indicating that the peak position was the same whereas the FWHM was slightly changed. In all cases, we confirmed that the correlation of the FWHM with the samples did not change. Since these parameters depend on the fitting model, we considered the relative differences of the peak position and FWHM in the discussion. We postulated a gaussian peak for the peak around 65 cm^−1^ because a gaussian function was used for fitting the main peak in the spectral analysis of the low-wavenumber region in the case of alkylimidazolium-based ILs and alkylammonium-based ILs [36,48].

## 4. Conclusions

Low-frequency Raman observation was performed with tetrabutylammonium and tetrabutylphosphonium salt hydrates as well as tetrabutylammonium and tetrabutylphosphonium salts. The spectra revealed that Raman peaks appeared in three wavenumber regions. In the range of 240–265 cm^−1^, Raman peaks due to a stretching lattice vibration mode in the cation were narrow and apparent in TBA salt hydrates and broad in TBP salt hydrates. In the range around 200 cm^−1^, Raman peaks, attributed to the O-O stretching vibration between water molecules, appeared below 200 cm^−1^, i.e., at a lower wavenumber in the TBAB·26H_2_O and TBAC hydrate. In the range around 65 cm^−1^, Raman peaks, affected by not only the water framework but the contribution of guest substances as well, were observed at a higher wavenumber in the TBAB·26H_2_O and TBAC hydrate. The trends in the peak shift were opposite in these two peaks. Furthermore, these two hydrates with higher equilibrium temperatures suggested that the equilibrium temperature in the semi-clathrate hydrates was related with the peak positions of the peak around 65 cm^−1^ and 200 cm^−1^. In designing a semi-clathrate hydrate, the direct observation of low-frequency Raman peaks could be used as an indicator of the equilibrium temperature of the hydrate.

## Figures and Tables

**Table 1:** Information on chemical compound.

Chemical Name	Source	Mass Fraction Purity
Tetrabutylammonium Bromide	Wako Pure Chemical Industries Ltd.	98+%
Tetrabutylammonium Chloride	Tokyo Chemical Industry Co. Ltd.	98%
Tetrabutylphosphonium Bromide	Wako Pure Chemical Industries Ltd.	95+%
Tetrabutylphosphonium Chloride	Iolitec Ionic Liquids Technologies GmbH	95+%
Ultrapure water	homemade by ADVANTEC RFU464TA	resistivity is 18.2 MΩ cm
Deuterium oxide	Cambridge Isotope Laboratories, Inc.	D, 99.9%

## Data Availability

The data presented in this study are openly available in figshare at 10.6084/m9.figshare.20364387.v1.
